# Gallstone ileus after laparoscopic cholecystectomy

**Published:** 2012-09-25

**Authors:** I Ivanov, M Beuran, MD Venter, I Iftimie-Nastase, R Smarandache, B Popescu, R Bostină

**Affiliations:** * Clinical Emergency Hospital, Bucharest, Romania; ** „Carol Davila” University of Medicine and Pharmacy, Bucharest, Romania

**Keywords:** gallstones, colon obstruction, calculus, intraperitoneal abscess, fistula

## Abstract

Gallstone ileus represents a rare complication (0,3-0,5%) of a serious, but common disease-gallstones, which affect around 10% of the population in the USA and Western Europe. Associated diseases (usually severe), elderly patients, delayed diagnosis and therapy due to late presentation to the hospital, account for the morbidity and mortality rates described in literature. We present the case of a patient with partial colon obstruction due to a large gallstone that was “lost” during an emergency laparoscopic cholecystectomy. The calculus eroded the intestinal wall, partially occluding the lumen, triggering recurrent Kerwsky-like, subocclusive episodes. The intraperitoneal abscess has spontaneously drained through the subhepatic drain and once the tube has been removed, a persistent intermittent fistula became obvious.

## Introduction

Gallstone ileus represents a rare complication (0,3-0,5%) [**1-4**] of a serious, but common disease-gallstones, which affect around 10% of the population in the USA and Western Europe [**5**]. Even today, when laparoscopic cholecystectomy is the “golden standard” of treatment, the incidence of this disease remains pretty much the same. Associated diseases (usually severe), elderly patients, delayed diagnosis and therapy due to late presentation to the hospital account for the morbidity and mortality rates described in literature.

Gallstone ileus accounts for 2-3% of all exploratory laparotomies for small bowel obstruction ([**6**], Kasahara cit. [**7**]) and in patients over 65 years of age, this type of ileus having an incidence of up to 25% of all unstrangulated bowel obstructions [**5**].

We present the case of a patient with partial colon obstruction due to a large gallstone that was “lost” during an emergency laparoscopic cholecystectomy. The calculus eroded the intestinal wall, partially occluding the lumen, triggering recurrent Kerwsky-like, subocclusive episodes. The intraperitoneal abscess has spontaneously drained through the subhepatic drain and once the tube has been removed, a persistent intermittent fistula became obvious.

## Case presentation

A 56 year-old female patient was admitted to the hospital for a 21-day-old onset of a colicky type of pain in her right abdominal flank and marked abdominal distension. Past medical history includes laparoscopic cholecystectomy 6 months prior, grade II hypertension, type II diabetes mellitus, and grade III obesity. The postoperative progress was rather difficult due to a small, purulent, drain discharge; the drain was removed in postoperative day 8. She is readmitted to hospital a month later with chronic right flank suppuration. The abdominal US is inconclusive (obese patient, abdominal distension, local postoperative alterations). Contrast was injected through the fistula and the X-ray revealed a 4 cm collection.

**Fig. 1:**
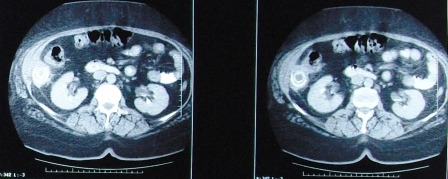
Abdominal CT scan shows a 50-60 mm, concentrically-layered, subhepatic collection with a dense, central, stone-like image, possibly sorrounded by the contrast material injected through the fistula; a segment VI hepatic abcess, exceeding the liver capsule, in direct contact with the walls of the right colonic angle and the ascending colon

**Fig. 2:**
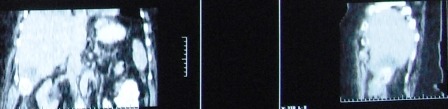
The same aspect- coronal section

**Fig. 3:**
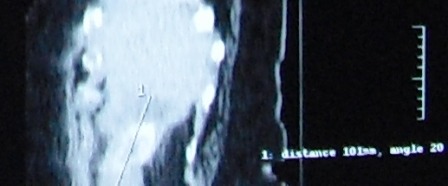
Abdominal CT showing the calculus and the subhepatic abscess (enhanced image)

The CT scan performed 5 months postoperatively revealed a 50-60 mm, concentrically-layered, subhepatic collection with a dense, central, stone-like image, possibly surrounded by the contrast material injected through the fistula; a segment VI hepatic abcess, exceeding the liver capsule, in direct contact with the walls of the right colonic angle and the ascending colon, which is displaced medially and anteriorly, and with the abdominal wall and the fistula on its right side (**Figures 1,2,3**).

**Fig. 4:**
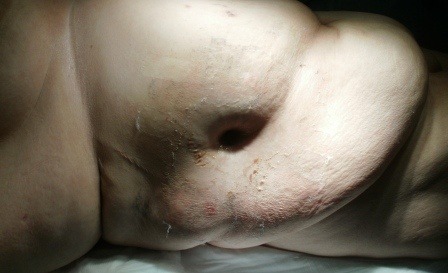
Cutaneous fistula upon admission

**Fig. 5:**
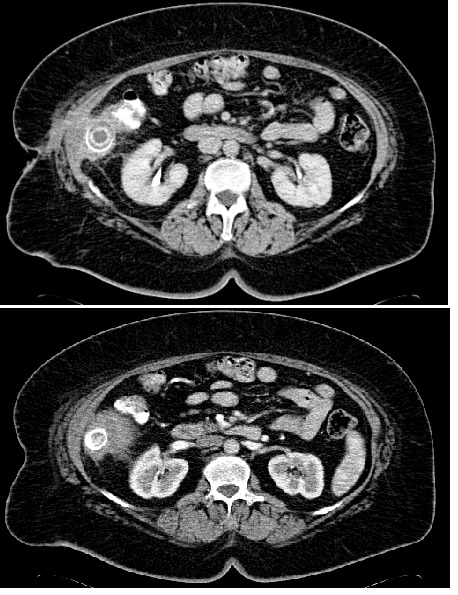
Abdominal CT upon admission- a central, round, partially calcified structure of about 3.2 cm; adjacent to this, there is a fistulous tract towards the skin

The physical exam upon admission revealed a patient in suffering, pyrexic but hemodinamically stable, with moderate abdominal distension and a right flank fistula with purrulent discharge (**Figures 4,5**).

The lab results showed: hemoglobin=10.3 g/dl, leucocytes=9600/cmm, hematocrit=31.5%, platelets=444000/cmm, glycemia=200 mg/dl, urea=10.6 mg/dl, total proteins=5.34 g/dl, albumin= 2.96 g/dl.

Double-contrast abdominal CT scan revealed a fatty liver, with a hypodense, non-homogenous, contrast-free area, of approximately 8/5,5 cm, situated in the lower part of the 6th segment, in direct contact with the ascending colon and with a central, round, partially calcified structure of about 3.2 cm. Adjacent to this, there was a fistulous tract towards the skin, a mild inflammatory edema around the lesion but not an abscess, gallbladder surgically removed. The conclusions stated that it appeared to be a subhepatic abscess, with a skin fistula, centered by what appeared to be a foreign body or calculus.

**Fig. 6:**
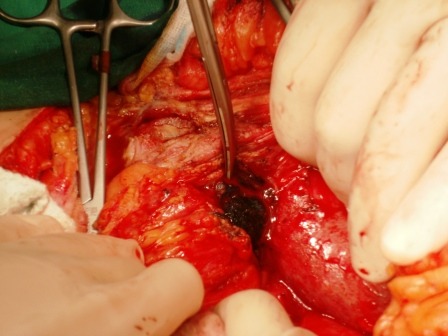
Intraoperatively: a 5/3 cm gallstone, partially lodged in the transverse colon (right flexure)

During surgery, a 5/3 cm calculus, partially occluding the transverse colon (the right angle) was found, as well as a sub-hepatic abscess (50 ml of pus) (**Figure 6**).

**Fig. 7:**
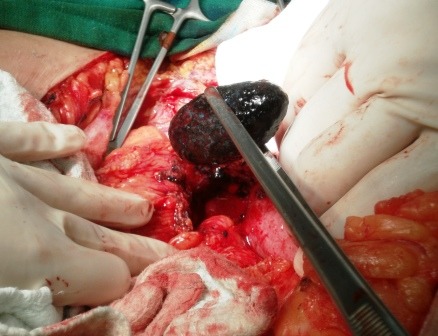
Intraoperatively: extracting the partially lodged stone from the colonic lumen

**Fig. 8:**
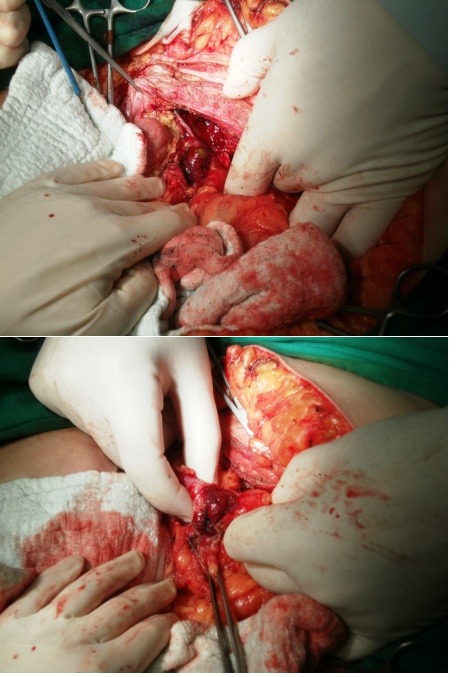
Colonic fistula

The gallstone is removed and a 1.5 cm long, colonic fistula is found (**Figures 7,8**). A fistulectomy is performed along with the suture of the colonic defect.

**Fig. 9:**
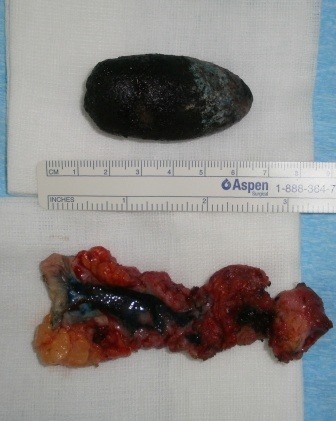
Surgical sample

The aspect of the operative specimen is shown in Figure 9.

The postoperative progress was excellent, the patient having been discharged in the postoperative day.

## Discussion

The gallstone ileus is a rare but severe complication of cholelithiasis. In order for this complication to occur, 3 conditions have been described as necessary:

1. a large gallstone (> 2.5 cm in diameter);

2. a cholecysto-digestive fistula should be present;

3. sometimes, the presence of a stenosed bowel.

However, these 3 conditions are not mandatory [**8**].

• The most frequent mechanism of gallstone ileus is represented by the migration of a gallstone through a cholecysto-duodenal fistula (68% of cases, 96.5% Japanese patients-[**9**]), but other types of fistulas have been reported as well: cholecysto-jejunal, cholecysto-colonic (5-25%- [**10,11**]), cholecysto-duodeno-colonic (2.5%), cholecysto-gastric (Clavien [**12**], Rodriguez Sanjuan [**13**], Glenn cit. [**14**]), choledocho-duodenal, choledocho-hepatic (left hepatic duct) [**1,15**].

Usually, the gallstone ileus is preceded by an episode of acute cholecystitis, followed by chronic inflammation and the development of adhesions between the intestines and the gallbladder (most of the times located at the fundic level). In addition to this, the ischemic and pressure effects triggered by the gallstone itself help form a bilio-digestive fistula (the reduction of arterial, venous and lymphatic flow associated with increased gallbladder pressure) [**1**]; sometimes the obstruction of the cystic duct is also associated [**16**]. The intestinal wall erosion is smoothed by the presence of crystallized biliary salts. In most cases, the gallbladder turns sclero-atrophic and rendered non-functional. Most frequently, the gallbladder fundus adheres to the duodenum, colon, stomach (in that order) and very rarely to the kidney, mesentery or to the liver itself.

The size of the calculus is also important in triggering the intestinal obstruction, most authors agreeing that a diameter >2.5 cm ensures complete intestinal occlusion (it is important that no other lumen stenosis is present caused by spasm, adhesions, Crohn’s disease). The surgically removed or naturally eliminated stones have average sizes ranging between 0.8-10 cm, 27% of patients exhibiting 4-5 cm gallstones. Bohan reports calculi weighing up to 465 g [**8**].

In 1975, after thoroughly analyzing 1000 cases of gallstone ileus described in literature, Day and Marks established that the average diameter is of 45 mm (ranging between 20 and 100 mm) and the weight of approximately 23.5 g (ranging between 4 and 68 g).

According to Suteu and Bucur [**17**], the main cause of gallstone ileus is represented by a dyssynergia between the spasm of the circular fibers and the hyperperistalsis of the longitudinal ones; the hyperperistalsis upstream aggravates intestinal lesions near the obstacle and at the occlusion site, the parietal edema could potentially lead to wall necrosis.

The calculus usually occludes the terminal ileum (65%) and the ileo-cecal valve (which is the most narrow segment of the small bowel and the peristalsis is reduced) [10,18,19]. The potentially active bile ingredients may interact with the intestinal cells and could induce complete occlusion and mucosal injury (Chipman cit. [**9**]).

The stone impaction is due not so much to mechanical factors but to bile-irritating mechanisms triggering the intramural paracrine signaling; at the blockage site a rapidly evolving edema ensues, which usually progresses to wall necrosis.

The sensitivity of the distal ileum to biliary salts is the main factor contributing to gallstone ileus, because the distal ileum traps most migrating foreign bodies, particularly those rich in bile salt [**17**].

According to Reisner [**18**], the colonic blockage usually occurs in 4% of cases, within the sigmoid colon (due to secondary stenosis following recurrent diverticulitis).

The colonic ileus most frequently occurs following a stone migrating through cholecysto-colonic fistula, as well as through a cholecysto-duodenal fistula (Moller 1913, Harris, McNamara si Dardinski 1947, Buetow, Glaubitz si Crampton 1963-cit. [**20**]) or a choledoco-duodenal fistula (Shore, Jacob and Cannon 1953). Holm-Nielsen and Linnet-Jepson consider that the ileus is due to progressive increase in initial stone size, while Haffner, Semb and Aakhus consider that the calculus size increases due to fecal accumulation around the stone [**20**]. The most frequent phenomenon is represented by the impaction of a large calculus in a spastic colon, while impaction secondary to lumen stenosis (recurrent diverticular disease, cancers) is less common.

The migration of a calculus through a fistula established between the common bile duct and the GI tract is uncommon [**21**].

• However, cases of gallstone intestinal obstruction without a bilio-digestive fistula have been described [**2,22**]. This is caused by the migration of a gallstone through the Vater papilla and its secondary enlargement within the intestine. Yoshida and co. have described a case of gallstone ileus after the calculus passed through the Vater papilla [**23**].

Lassandro [**24**], Lindsey and Warner (cit. [**25**]) have described a case of gallstone ileus after cholecystectomy, the calculus having migrated along the common bile duct (after sphyncterotomy) or from a pulsion duodenal diverticulum. Saedon [**26**] reports a case of gallstone ileus 24 years after cholecystectomy, in a patient with jejuno-ileal diverticular disease.

Draganic [**25**] and Dittrich [**27**] report two cases of intestinal obstruction when a “lost” calculus after a difficult laparoscopic cholecystectomy migrated through the jejunal wall. Wills [**28**] described a case of partial intestinal obstruction due to gallstones, that was spontaneously resolved, but Dragnic and Dittrich were the first to publish articles referring to gallstone ileus. Other authors reported cases with the similar mechanism [**29**].

Habib [**30**] reported a case of gallstone ileus occurring 8 years after laparoscopic cholecystectomy during which a calculus was “lost” in the abdominal cavity; the stone migrated through the greater omentum, eroded the apex of a Meckel diverticulum, lodging at its base, eventually migrating into the intestinal lumen, occluding it. 

Beltran [**31**] suggested another possible mechanism represented by an association between a Mirizzi syndrome and cholecysto-enteral fistula, other authors having described cases of gallstone ileus in patients presenting type IV Mirizzi syndrome and cholecysto-colonic fistula.

A particular place is granted to patients with Crohn’s disease, who after a long progress, develop gallstones. This phenomenon might be explained by altered biliary cholesterol solubility and its cholesterol precipitation (due to altered bowel-liver circulation), increased bilirubin concentration in the bile, which can determine enlargement of pigmentary calculi due to enteral motility imbalances [**32-34**].

All injuries to the gallbladder during laparoscopic cholecystectomy usually occur during its dissection or its extraction from the abdomen; in up to 20% of cases, lost intraperitoneal calculi are not retrieved because of their great number, their friability or their localization within the peritoneal cavity (Targarona-[**35**]).

Lost intraperitoneal calculi may cause abscesses, fistula, and pseudo-tumoral inflammatory reactions, or could erode the neighboring hollow organs, particularly if the size is significant or are fragmented or pigmentary. Associated infected bile may also be a risk factor for this condition to occur (Sax-[**36**]).

Clinical studies have shown an incidence of complications triggered by lost calculi ranging between 0,5 – 6% of all laparoscopic cholecystectomies.

If the abscess or the gallstone are adjacent to a drain or a trocar site, a persistent external fistula may ensue (which is the situation described in this paper). Intestinal wall erosion (small bowel or colon) by a large calculus may produce gallstone ileus.

The definitive diagnosis is difficult and relies on CT scan results, showing an ectopic gallstone and radiological signs of intestinal obstruction, without pneumobilia. It is helpful to first needle-aspirate the gallbladder once in the abdominal cavity and gently handled by non-traumatic graspers; whatever accidentally lost calculi should be retrieved by using an endo-bag and, an enlargement of the trocar incision may be necessary as well to prevent accidental bag rupture. It is highly recommended to laparoscopically extract all intraperitoneal calculi, particularly if their size is great, they are pigmentary, fragmented or if the bile is infected [**37,38**]. It is also necessary to inform the patient and to ensure adequate follow-up by ultrasound and clinical monitoring for an early diagnosis of septic or obstructing complications (Habib [**30**]).

We believe the case presented in this paper is the first case of gallstone ileus associated with a persistent external fistula occurring after laparoscopic cholecystectomy. It also underlines the role of the Clinical Emergency Hospital in the acute, non-traumatic surgery.

This case presentation, the first of its kind in the Romanian medical literature, describes a case of an incomplete gallstone ileus after cholecystectomy in the absence of a “classical” billio-digestive fistula, where “lost” calculus eroded the colonic wall.
